# Donation Parties

**Published:** 1876-06

**Authors:** 


It is not in human nature to resist the mollifying influences of gifts: the great mind of Lord Bacon could not resist it; hence it is every where ground for impeachment and removal as to our judges, and there is a universal sentiment of approval, even in the midst of the most uncultivated of our population. In short, there is only one true, honorable, safe and manly course to be pursued in reference to our clergy, and if it were universally adopted, it would throw over this nation a conservative and an elevating influence which would make it in reality the glory of the whole earth; and it is this: Let every evangelical clergyman of all sects have a salary fully adequate to a plain, unostentatious mode of life, and let it be promptly, regularly and fully paid in money, to the very last cent; and never let him be presented with a single dollar or its value, except under circumstances which would effectually prevent the giver being known, except to that giver and to God, who will reward him openly in due time.

